# An Adaptive Octile JPS and Fuzzy-DWA Fused Path Planning Algorithm for Indoor Home Environments

**DOI:** 10.3390/s26113300

**Published:** 2026-05-22

**Authors:** Wei Li, Zhuoda Jia, Dawen Sun, Deng Han, Zhenyang Qin, Qianjin Liu

**Affiliations:** 1 College of Mechanical and Vehicle Engineering, Changchun University, Changchun 130022, China; liw82@ccu.edu.cn (W.L.); jzd1604712294@163.com (Z.J.); qin02666@163.com (Z.Q.); liu_qj0405@163.com (Q.L.); 2 Key Laboratory of Intelligent Rehabilitation and Barrier-Free Education for Disabled Persons, Ministry of Education, Changchun 130022, China; 3 College of Computer Science and Technology, Changchun University, Changchun 130022, China; 4 Jilin Province Zhixing Internet of Things Research Institute Co., Ltd., Changchun 130022, China; handeng163@163.com

**Keywords:** path planning, jump point search, look-ahead distance, dynamic window approach, indoor navigation

## Abstract

Home indoor environments are characterized by alternating open spaces and obstacle-cluttered regions, which pose critical challenges to the autonomous navigation of home service robots. Existing hybrid path planning algorithms generally suffer from three core limitations: low global search efficiency, weak global-local planning coordination, and poor dynamic scene adaptability. To tackle these issues, this paper presents a novel hierarchical path planning framework combining an enhanced Jump Point Search (JPS) and a fuzzy-optimized Dynamic Window Approach (DWA). In the global planning layer, an adaptive Octile heuristic JPS based on local obstacle density is designed to reduce redundant node expansion and accelerate global path search, with a bounded suboptimality guarantee. To bridge global and local planning, a look-ahead distance-based dynamic waypoint selection strategy is developed to match the optimal waypoint in real time according to the robot’s motion state and environmental complexity, enabling seamless coordination between global path guidance and local trajectory generation. In the local planning layer, a fuzzy logic controller is introduced to dynamically tune the weights of the DWA trajectory evaluation function, which significantly improves the robot’s dynamic obstacle avoidance capability and motion smoothness. Comparative simulation experiments verify that the proposed method not only outperforms the conventional hybrid path planning algorithm, reducing expanded nodes by 68.09% and global planning time by 52.94%, while improving dynamic obstacle avoidance success rate by 31.43% and overall navigation efficiency by 23.95%, it also achieves better comprehensive navigation performance than the widely adopted PSO-DWA comparison algorithm. The proposed framework shows superior comprehensive performance and is well suited for the indoor autonomous navigation of home service robots.

## 1. Introduction

With the widespread deployment of home service robots in applications including in-home companionship and intelligent cleaning, autonomous navigation has become the core functionality of such systems, and path planning serves as the fundamental cornerstone for enabling reliable autonomous navigation. The core objective of path planning is to generate a collision-free, optimal path from a start point to a target point for the robot, which adheres to the robot’s kinematic constraints while satisfying spatial and obstacle constraints in indoor home environments. According to the spatial scope of planning and reliance on environmental information, path planning algorithms are broadly categorized into two classes: global path planning and local path planning.

Global path planning generates a globally collision-free, optimal path for the robot given a pre-known global static environment map, with the core objective of ensuring the global optimality and feasibility of the planned path. Such algorithms perform computations relying solely on a pre-constructed global map, without requiring real-time environmental perception data, making them well-suited for static indoor scenarios with fixed layouts. Typical global path planning algorithms include the Dijkstra algorithm [1], the A* algorithm [2], and the Jump Point Search (JPS) algorithm [3], among others. Of these, the JPS algorithm eliminates a large number of redundant expanded nodes inherent to the A* algorithm via jump point pruning rules, significantly improving search efficiency while strictly guaranteeing path optimality. This makes it one of the most widely adopted global planning algorithms for indoor static scenarios.

Local path planning dynamically adjusts the robot’s short-term motion trajectory during navigation, based on real-time perceived local environmental information, with the core objective of achieving real-time avoidance of sudden dynamic obstacles. Such algorithms do not require prior full knowledge of the environment and can adapt to dynamically changing scenarios. However, limited by the range of local perception, they are prone to falling into local optima or even suffering from planning failure. Typical local path planning algorithms include the Artificial Potential Field (APF) method [4], the Timed Elastic Band (TEB) method [5], and the Dynamic Window Approach (DWA) [6], among others. Of these, the DWA algorithm generates a feasible velocity window based on the robot’s kinematic constraints and selects the optimal velocity command via a multi-objective cost function. It features high computational efficiency and strong robustness, making it the mainstream algorithm for local obstacle avoidance in robotic systems.

The hierarchical fused global-local path planning architecture, which combines the global optimality of global planning and the real-time obstacle avoidance capability of local planning, can simultaneously satisfy the core requirements of globally optimal collision-free paths, real-time local dynamic obstacle avoidance, and smooth, kinematically feasible trajectory. It has thus become the mainstream technical solution for the indoor navigation of home service robots. Aiming at the navigation requirements of indoor service robots, researchers worldwide have conducted extensive research on three core directions of this fused architecture: optimization of the global planning module, improvement of the local planning module, and design of the global-local coordination strategy, forming a well-established research framework.

In the direction of global planning module optimization for the fused architecture, the JPS algorithm has become the most widely used global planning algorithm in fused solutions, owing to its high search efficiency and strict theoretical guarantee of path optimality. Fan et al. introduced a passable value weight and improved filtering criterion into the JPS heuristic function and combined the Akima interpolation algorithm to generate a safe and smooth path [7]. Sun et al. improved the JPS algorithm via a density-aware heuristic function, key point extraction, and B-spline fitting, enhancing planning efficiency and tracking stability [8]. Luo et al. proposed the AP-JPS algorithm combined with an angle propagation algorithm to optimize the practical adaptability of paths in discrete grid maps [9]. Li et al. adopted a selective bidirectional search strategy in their improved algorithm, which prioritizes in-depth search toward the target direction, effectively reducing the number of jump point selections, shortening pathfinding time, and significantly improving global path planning efficiency [10]. Guo et al. designed an improved bidirectional dynamic jump point search algorithm, which optimizes node expansion by introducing an adaptive weight coefficient into the heuristic function and combining a dynamic constraint circle, thus significantly reducing search time and improving the efficiency and safety of path planning for mobile robots [11]. Fan proposed a target-optimized jump point search method, which constructs search direction priorities based on the direction vector of the target point and improves the cost function, effectively reducing redundant nodes, cutting computation time, and achieving superior path quality [12]. Hu et al. integrated a heuristic function combining direction angle and weight factor with a bidirectional JPS architecture and combined a path pruning method to improve search efficiency and path smoothness while ensuring path safety [13]. However, most existing JPS optimizations in fused solutions focus on the distance constraint between the node and the target point, and rarely achieve adaptive adjustment of the heuristic function by incorporating the scenario characteristics of local obstacle distribution. In home indoor scenarios with alternating open areas and obstacle-dense regions, these methods still suffer from redundant global search and slow planning speed, which directly degrade the real-time performance of the fused navigation system.

In the direction of global-local coordination strategy design for the fused architecture, waypoint selection is the core link that determines the coordination performance of the fused navigation system. Oriolo et al. pioneered a rolling window-based waypoint selection method, establishing the classic closed-loop navigation framework of global mapping and planning, waypoint coordination, and local tracking execution in unknown environments [14]. Wang et al. tightly coupled global and local planning algorithms via a waypoint guidance mechanism, which ensures high-quality global path planning while overcoming the inherent limitations of traditional local planning [15]. Su et al. adopted global key point extraction and inflection point-guided segmented local planning, which effectively reduced planning time and the number of inflection points and improved path smoothness and local obstacle avoidance capability [16]. Jian constructed a sampling evaluation system integrating prior information of the global optimal solution and designed a new local path cost function to strengthen global guidance constraints, which effectively improved the coupling matching degree between the global path and local trajectory [17]. Liao et al. introduced the Douglas–Pucker algorithm to retain feature points on frequently curved paths, which effectively reduced redundant nodes and search time and improved path smoothness and adaptability to dynamic and static environments [18]. Zhang et al. proposed a key node selection strategy that optimizes node selection and eliminates redundant nodes, effectively improving path smoothness and environmental adaptability [19]. However, most existing waypoint screening methods in fused solutions are designed with fixed rules or based solely on the geometric characteristics of the path and thus rarely achieve dynamic adjustment of the look-ahead distance by combining the obstacle distribution of the scenario and the motion state of the robot. In complex indoor home scenarios, these methods still suffer from unreasonable waypoint selection and insufficient global-local coordination; in severe cases, the local trajectory may deviate from the globally optimal path.

In the direction of local planning module improvement for fused architecture, the DWA is the preferred algorithm for local obstacle avoidance and trajectory tracking in fused solutions, due to its high computational efficiency and strong robustness. Chai et al. improved the DWA algorithm by adding target distance and safety distance cost functions to avoid the local optimum problem [20]. Wang et al. adaptively adjusted the weights of the DWA evaluation function via a fuzzy logic controller, realizing multi-objective optimization of obstacle avoidance, efficiency, and smoothness [21]. Gong et al. improved the velocity and target distance cost functions of DWA and obtained adaptive weights combined with fuzzy control, which enhanced obstacle avoidance capability in complex environments [22]. Wang et al. integrated the Deep Deterministic Policy Gradient (DDPG) algorithm with DWA, realizing self-learning of obstacle avoidance strategies via reinforcement learning [23]. Yan et al. added a distance scoring mechanism between the current position and the target point to optimize the performance of DWA in obstacle-dense areas [24]. Xiao et al. combined an improved ant colony algorithm with DWA to improve the path quality of indoor navigation via global planning and local optimization [25]. Li et al. integrated an improved Particle Swarm Optimization (PSO) algorithm with DWA to meet the adaptability requirements of local path planning [26]. However, most existing DWA improvements in fused solutions focus on the balance between obstacle avoidance safety and tracking accuracy and rarely realize dynamic weight adjustment by combining the characteristics of the global waypoint. When sudden scenario changes occur in indoor home environments, these methods still suffer from lagging weight adjustment and insufficient scenario adaptability, resulting in a low success rate of local obstacle avoidance for the fused navigation system.

Meanwhile, data-driven path planning methods have also gained widespread attention. Chen et al. [27] proposed a convolutional neural network (CNN)-based foothold selection method that enables energy-efficient locomotion of quadruped robots on complex terrains. In addition, reinforcement learning (RL)-based methods can autonomously learn obstacle avoidance strategies through interaction with the environment, demonstrating excellent adaptability in dynamic and complex scenarios [28]. However, data-driven methods typically require large amounts of labeled data or prolonged interactive training and suffer from inherent limitations, including poor interpretability, limited generalization ability, and high computational resource demands, making them difficult to be directly deployed on embedded platforms of home service robots with constrained computing resources.

To clearly delineate the core innovations of this work from prior studies, Table 1 presents a systematic comparison between the proposed method and the most representative recent works in the three core research directions of the fused architecture. The comparison is conducted from three dimensions: global planning heuristic design, global-local coordination mechanism, and local planning weight adjustment.

As summarized in Table 1, existing works typically focus on optimizing one or two of the three core dimensions, while the remaining dimensions are either not addressed or handled with conventional fixed-rule approaches. Specifically, in global planning, existing density-aware heuristics adopt global density statistics and Euclidean distance bases, lacking local perception granularity and formal admissibility guarantees. In global-local coordination, existing waypoint selection methods rely predominantly on fixed rules or path geometry characteristics, failing to dynamically adapt to scene complexity and robot motion state. In local planning, existing DWA improvements seldom couple the fuzzy controller inputs with local waypoint characteristics, limiting the scene adaptability of weight tuning.

To address the above limitations, this paper proposes a hierarchical path planning algorithm integrating an improved JPS and a fuzzy-optimized DWA for home service robots. The algorithm adopts a three-layer fused architecture based on the principle of “global guidance, local execution,” where the upper global planning layer provides local perception granularity and formal bounded-suboptimality guarantees through an adaptive Octile heuristic, the intermediate coordination layer achieves dynamic global-local adaptation through a look-ahead distance-based waypoint selection strategy, and the lower local planning layer enhances scene-adaptive weight tuning through a fuzzy controller coupled with local waypoint characteristics.

## 2. Design of the Adaptive JPS-Fuzzy DWA Fused Path Planning Algorithm

### 2.1. Overall Framework of the Proposed Hierarchical Fused Algorithm

**Figure 1:**
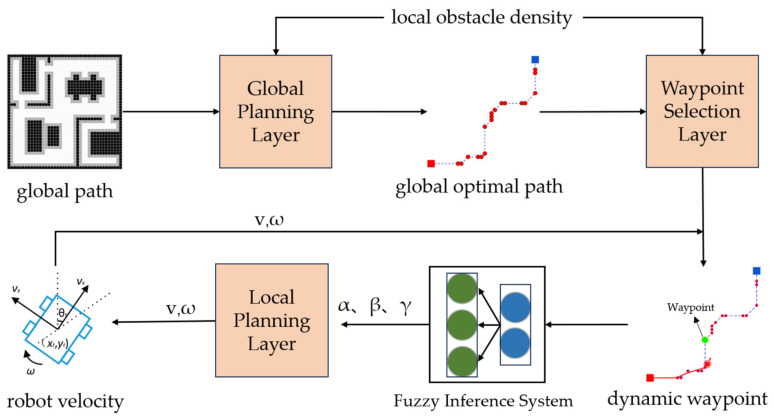
Overall framework of the proposed hierarchical fused path planning algorithm.

The overall framework of the path planning algorithm proposed in this paper is shown in Figure 1, and its core idea is a hierarchical fusion strategy based on the principle of “global guidance, local execution.” The algorithm architecture consists of three tightly coupled layers, as detailed below:

1. Upper-layer global planning module: Centered on the improved adaptive heuristic JPS algorithm, this module generates a globally optimal path from the start point to the target point based on a known static global map, addressing the inherent limitations of redundant node expansion and low search efficiency in the conventional JPS algorithm.
2. Intermediate global-local coordination module: Built upon the look-ahead distance-based dynamic waypoint selection strategy, this module matches the optimal waypoint for local planning in real time from the global jump point sequence, according to the complexity of the current scenario and the real-time motion state of the robot. It solves the core problem of insufficient coordination between global path guidance and local trajectory planning in traditional fused schemes.
3. Lower-layer local planning module: Focused on the fuzzy control-optimized DWA, this module takes the currently matched waypoint as the tracking target, generates optimal velocity commands that satisfy the robot’s kinematic constraints based on real-time perceived local environmental information, and achieves reliable dynamic obstacle avoidance and high-precision trajectory tracking. It eliminates the defects of fixed weight allocation and poor scenario adaptability in the conventional DWA algorithm.

### 2.2. Adaptive Octile Heuristic JPS Algorithm Based on Local Obstacle Density

The standard JPS algorithm is an optimized graph search algorithm derived from the A* framework. Its core advantage is that it eliminates a large number of redundant expanded nodes in the A* algorithm via predefined jump point pruning rules and only retains key jump points with forced neighbors. This mechanism greatly reduces the search space scale while strictly guaranteeing the optimality of the planned path.

The node prioritization rule of JPS is consistent with that of the A* algorithm, where nodes are sorted by priority according to the cost function f(n):

(1) $$f\left(n\right)=g\left(n\right)+h\left(n\right)$$

where g(n) denotes the actual accumulated cost from the start point to the current node n, and h(n) represents the heuristic estimated cost from the current node n to the target point.

For the eight-directional grid motion model widely used in indoor mobile robots, the standard JPS adopts the Octile distance as the heuristic function, which is formulated as follows:

(2) $$h_{\mathrm{octile}}\left(n\right)=\max(d_{x},d_{y})+(\sqrt{2}-1)\cdot \min(d_{x},d_{y})$$

where d_x_ and d_y_ denote the absolute values of the row and column coordinate differences between the current node and the target point, respectively.

The core limitation of the standard JPS is that the fixed-form Octile heuristic cannot adaptively adjust the guidance strength according to the obstacle distribution of the local scenario. In home indoor scenarios with alternating open areas and obstacle-dense regions, insufficient guidance in open areas leads to massive redundant node expansion, while excessive guidance in dense regions causes search oscillation, ultimately resulting in low global planning efficiency.

Existing studies have attempted to incorporate obstacle density to optimize heuristic functions, but these methods suffer from two fundamental limitations. First, they directly use obstacle density as a heuristic multiplier, which cannot guarantee bounded suboptimality of the heuristic function and may result in unbounded degradation of global path optimality. Second, they predominantly adopt global obstacle density statistics, failing to achieve fine-grained perception of local scene variations.

To address the above limitations, this paper designs an adaptive Octile heuristic function based on local obstacle density. With a bounded-suboptimality guarantee, the proposed function dynamically adjusts the heuristic weight according to the local scenario characteristics around the node, so as to improve global search efficiency without incurring unbounded path degradation.

The formula of the designed adaptive heuristic function is expressed as

(3) $$h\left(n\right)=h_{\mathrm{octile}}\left(n\right)\cdot \left[1+\alpha \cdot \left(1-\rho \left(n\right)\right)\right]$$

where α is the pre-defined heuristic weighting coefficient, and ρ(n) denotes the local obstacle density within the neighborhood of node n.

In a grid map with an eight-directional movement model, the true optimal cost from any node n to the goal satisfies the inherent bound:

$$h_{\mathrm{octile}}\left(n\right)\leq h*\left(n\right)\leq \sqrt{2}h_{\mathrm{octile}}\left(n\right)$$

To limit the maximum amplification factor of the heuristic and avoid excessive overestimation beyond this theoretical bound, the maximum heuristic weight is constrained by $1+\alpha \leq \sqrt{2}$, which gives the feasible range $\alpha \in \left[0,\sqrt{2}-1\right]$.

Hence, the proposed adaptive heuristic satisfies

$$h\left(n\right)\leq h_{\mathrm{octile}}\left(n\right)\cdot \left(1+\alpha \right)\leq h_{\mathrm{octile}}\left(n\right)\cdot \sqrt{2}$$

Together with $h_{\mathrm{octile}}\left(n\right)\leq h*\left(n\right)$, we further obtain

$$h\left(n\right)\leq \sqrt{2}h*\left(n\right)$$

According to the classic theory of weighted A* search [29,30], if a heuristic satisfies $h\left(n\right)\leq \omega h*\left(n\right)$, then the cost C of the path returned by the algorithm is bounded by C≤ωC*, where C* is the true optimal cost. Therefore, the proposed adaptive heuristic is a bounded-suboptimal heuristic with a theoretical upper bound of $\sqrt{2}$ times the optimal cost.

- In obstacle-dense regions where $\rho \left(n\right)\to 1$, the adaptive weight approaches 1, so $h\left(n\right)=h_{\mathrm{octile}}\left(n\right)$. This strictly satisfies the admissibility condition $h\left(n\right)\leq h*\left(n\right)$, avoiding any overestimation and preserving search rigor in complex areas.
- In medium-density or free regions where $0\leq \rho \left(n\right)<1$, the adaptive weight lies between 1 and 1 + α. The heuristic may no longer be strictly admissible, but the overestimation is controlled and always satisfies $h\left(n\right)\leq \sqrt{2}h*\left(n\right)$. By the weighted A* theory, this guarantees that the final path cost does not exceed $\sqrt{2}$ times the optimum. Moreover, because the overestimation is mild and the JPS pruning preserves most of the optimality, experiments show that the returned path is almost always optimal. This design enhances goal-directedness, effectively reduces redundant jump points and node expansions, and significantly improves global search efficiency while providing a bounded-suboptimality guarantee.

### 2.3. Look-Ahead Distance-Based Dynamic Waypoint Selection Strategy

The jump point path generated by the global JPS module is a discrete sequence of key nodes. If all jump points are directly adopted as the tracking waypoints for local path planning, it will result in excessively frequent waypoint switching, which forces the robot to perform frequent deceleration and steering maneuvers. This, in turn, gives rise to oscillatory and non-smooth motion trajectories and ultimately degrades the overall navigation efficiency. By contrast, the fixed-interval point sampling method fails to adapt to variations in scene complexity; it tends to cause unreachable waypoints in narrow obstacle-dense areas and suffers from overly dense waypoints in open scenarios.

**Figure 2:**
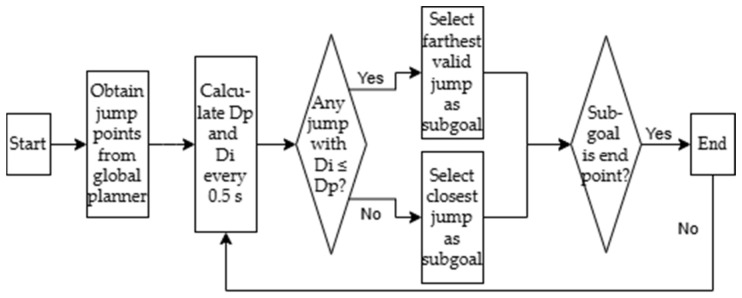
Flowchart of the waypoint selection strategy. Dp: robot’s look-ahead distance; Di: distance between current position and jump point.

To address the above challenges, this paper proposes a dynamic waypoint selection strategy based on adaptive look-ahead distance, whose execution workflow is illustrated in Figure 2. The core principle of the proposed strategy is that the look-ahead distance threshold is dynamically adjusted in real time according to the environmental complexity of the robot’s operating scene and its real-time motion state, so as to select the optimal waypoint matching the robot’s local planning capability from the global jump point sequence. This mechanism ensures that waypoint selection can always adapt to the real-time motion characteristics of the robot and the constraints of the surrounding environment, ultimately achieving smooth coupling and coordinated operation between global path guidance and local path planning.

The look-ahead distance D_p_ is defined as the maximum guiding distance that the robot can stably track without deviating from the global path under its current motion state and ambient environment, with its calculation formula given by

(4) $$D_{p}=D_{b}\cdot \frac{v_{cur}}{v_{\max}}\cdot \frac{1}{1+\rho }$$

where D_b_ denotes the pre-defined base look-ahead distance, v_cur_ is the current linear velocity of the robot, v_max_ represents the maximum allowable linear velocity of the robot, and ρ is the local obstacle density of the robot’s current position.

### 2.4. Fuzzy Control-Based Optimization of the Dynamic Window Approach

The standard DWA algorithm generates a feasible velocity window based on the robot’s kinematic constraints and selects the optimal motion trajectory of the robot at the current time step through a multi-objective trajectory evaluation function. Essentially, it selects the optimal solution from the velocity candidate set under the premise of satisfying both the robot’s kinematic constraints and environmental safety constraints.

Suppose the robot has a linear velocity v_t_ and an angular velocity ω_t_ at time t. Let v_max_ denote the maximum linear velocity, v_min_ the minimum linear velocity, a_max_ the maximum linear acceleration, and α_max_ the maximum angular acceleration of the robot. Then, the feasible velocity set v_d_ of the robot within the future time interval Δt is expressed as

(5) $$v_{d}=\left\{\left(v,\omega \right)\left|\begin{array}{l} v\in \left[\max\left(v_{\min},v_{t}-a_{\max}\Delta t\right),\min(v_{\max},v_{t}+a_{\max}\Delta t)\right]\\ \omega \in \left[\max\left(\omega_{\min},\omega_{t}-\alpha_{\max}\Delta t\right),\min(\omega_{\max},\omega_{t}+\alpha_{\max}\Delta t)\right] \end{array}\right.\right\}$$

After velocity sampling, multiple reachable motion trajectories of the robot can be obtained via dead reckoning equations. Each trajectory is scored by the multi-objective evaluation function, and the velocity corresponding to the trajectory with the highest score is selected as the optimal output. The evaluation function of the standard DWA is given by

(6) $$G\left(v,\omega \right)=\alpha head\left(v,\omega \right)+\beta dist\left(v,\omega \right)+\gamma vel\left(v,\omega \right)$$

where

- head(v,ω) is the heading evaluation term, which measures the heading deviation between the endpoint of the predicted trajectory and the target waypoint; a larger value indicates better heading alignment;
- dist(v,ω) is the obstacle avoidance evaluation term, which measures the minimum distance between the predicted trajectory and the nearest obstacle, with a larger value indicating higher obstacle avoidance safety;
- vel(v,ω) is the velocity evaluation term, which measures the linear velocity magnitude of the predicted trajectory, with a larger value indicating higher navigation efficiency;
- α, β, and γ are the fixed weighting coefficients of the three evaluation terms, with the normalization constraint α + β + γ = 1.

The conventional DWA algorithm adopts fixed weights for the evaluation function, which cannot be dynamically adjusted in real time with scene variations. This leads to the inability to prioritize tracking accuracy and navigation efficiency in open scenarios, as well as the failure to guarantee obstacle avoidance safety in obstacle-dense areas, resulting in insufficient scene adaptability and flexibility.

To address the above limitations, this paper introduces a fuzzy control module to realize adaptive weight tuning for the DWA trajectory evaluation function. The module takes two variables as inputs: the minimum distance between the robot and the nearest obstacle, and the heading deviation between the robot and the target waypoint. The outputs are three weighting coefficients: α (heading tracking weight), β (obstacle avoidance weight), and γ (velocity weight).

The membership functions, universe of discourse, and corresponding fuzzy sets for each variable are defined as follows:

- Input 1: Heading deviation. This variable describes the angular difference between the robot’s current heading and the direction to the target waypoint, with its universe of discourse set to [0, π]. The corresponding fuzzy sets are {Small (S), Medium (M), Large (L)}, with triangular membership function vertex coordinates S (0, 0, π/3), M (π/6, π/2, 5π/6), and L (2π/3, π, π). This design ensures the target waypoint always lies within the robot’s forward navigation range.
- Input 2: Minimum distance. This variable characterizes the real-time safety margin between the robot and surrounding obstacles, with its universe of discourse set to [0, 3]. The corresponding fuzzy sets are {Near (N), Medium (M), Far (F)}, with triangular membership function vertex coordinates N (0, 0, 1.8), M (0.8, 1.5, 2.2), and F (1.8, 3, 3). This design ensures clear differentiation of obstacle proximity levels in typical indoor home environments, allowing the robot to implement graded avoidance strategies according to the real-time safety margin.
- Output: Weighting coefficients. The universe of discourse for each coefficient is set to [0, 1], with fuzzy sets {Small (S), Medium (M), Large (L)}. All three output variables share identical triangular membership function parameters: S (0, 0, 0.4), M (0.2, 0.5, 0.8), and L (0.6, 1, 1). This design enables continuous adaptive tuning of the relative importance of heading tracking, obstacle avoidance, and navigation efficiency.

**Figure 3:**
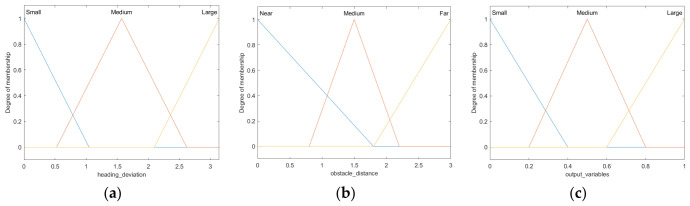
Membership function curves of the input and output variables for the designed fuzzy logic controller: (**a**) membership functions for the input variable: heading deviation to the target waypoint; (**b**) membership functions for the input variable: minimum distance to the nearest obstacle; (**c**) membership functions for the output variables: DWA trajectory evaluation function weighting coefficients α, β, γ.

To balance computational efficiency and control smoothness, all fuzzy sets adopt triangular membership functions with appropriate overlap between adjacent sets. This ensures that at least one fuzzy set is activated for any input value within its universe of discourse, thus eliminating potential control blind spots. The membership function curves of all variables are presented in Figure 3.

The design of fuzzy rules strictly follows the core logic of mobile robot path planning:

- When the robot is close to an obstacle, the obstacle avoidance weight β is preferentially increased while the velocity weight γ is reduced, to prioritize navigation safety.
- When the robot is in an open obstacle-free area, the velocity weight γ is preferentially increased while the obstacle avoidance weight β is reduced, to prioritize navigation efficiency.
- When the heading deviation between the robot and the target waypoint is large, the heading weight α is preferentially increased, to ensure the trajectory is oriented toward the target and improve tracking accuracy.

Based on the above design principles and physical significance, the dual-input three-output fuzzy rule table is given as Table 2.

This paper employs the Mamdani-type fuzzy inference method for fuzzy logic operations, where fuzzy implication uses the minimum operation and fuzzy aggregation uses the maximum operation. To obtain precise control outputs, the Centroid Method is adopted for defuzzification. This method utilizes the information from all activated rules to generate continuous and smooth control signals, eliminating control step jumps. It is particularly suitable for mobile robot control scenarios that require high motion stability.

**Figure 4:**
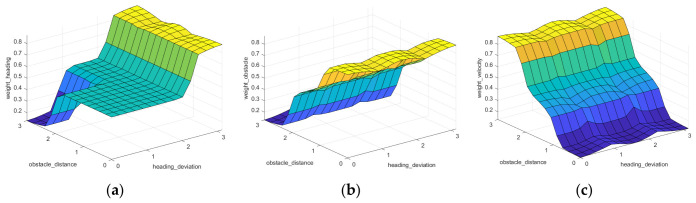
Full input-domain input-output mapping surfaces of the proposed dual-input three-output fuzzy logic controller: (**a**) mapping surface for the output heading tracking weight α; (**b**) mapping surface for the output obstacle avoidance weight β; (**c**) mapping surface for the output velocity regulation weight γ.

To verify whether the input-output mapping characteristics of the designed dual-input three-output fuzzy logic controller align with the control logic of mobile robot path planning, a full input-domain visualization analysis of the Fuzzy Inference System is conducted, with the results presented in Figure 4.

As can be observed from the full input-domain mapping surfaces in Figure 4., the three output weights of the designed fuzzy controller maintain smooth and continuous variation characteristics across the full operating range, with no step jumps in control variables or output breakpoints.

Meanwhile, the three weights exhibit a significant complementary adaptive adjustment law. They dynamically adjust the weight proportion of each sub-term in the path evaluation function according to the robot’s real-time heading deviation from the target waypoint and distance to the nearest obstacle. Through the dynamic trade-off between weights, multi-objective balance and collaborative optimization of safe obstacle avoidance, efficient navigation, and high-precision heading tracking are achieved. These results fully verify the rationality and engineering effectiveness of the designed dual-input three-output fuzzy controller.

## 3. Simulation Experiments

### 3.1. Experimental Setup and Parameter Configuration

To systematically verify the effectiveness and superiority of the proposed fused path planning algorithm integrating the improved JPS and fuzzy control-optimized DWA, an experimental framework based on a single-module ablation study is adopted in this work. Algorithm validation is conducted from three core dimensions: global search efficiency, global-local collaborative adaptability, and scene adaptability of local path planning.

All simulation experiments are implemented on the MATLAB R2021a platform. A 40 × 40 grid map model is constructed based on a typical indoor home scenario, which covers typical features of home environments including narrow corridors, room entrances and exits, furniture-dense obstacle areas, and open traffic aisles. This experimental setup enables comprehensive testing of the algorithm’s robustness and scene adaptability in complex indoor environments.

A two-wheeled differential drive kinematic model, which is widely adopted for home service robots, is used in the experiments. The core motion parameters are set in accordance with the actual operating characteristics of commercial household robots, with detailed specifications listed in Table 3.

### 3.2. Sensitivity Analysis of Key Parameters

To verify the robustness of the proposed algorithm to key parameters and to justify the parameter selection, a systematic sensitivity analysis is conducted on the four core parameters that have the most significant impact on algorithm performance. The control variable method is adopted, where only one parameter is changed at a time while all other parameters are kept at their default values. All experiments are repeated 50 times, and the results are presented as mean values.

The heuristic weight α controls the maximum amplification factor of the adaptive heuristic in open areas. According to the theoretical derivation in Section 2.2, α must satisfy $\alpha \leq \sqrt{2}-1$ to guarantee heuristic admissibility. The value of α is tested in the range of 0.10 to 0.40, and the global planning time, number of expanded nodes, and path length are recorded. The results are presented in Table 4.

As α increases from 0.10 to 0.40, the global planning time decreases monotonically from 31.8 ms to 21.5 ms, and the number of expanded nodes decreases from 42 to 25, indicating a continuous improvement in search efficiency. However, when α exceeds 0.25, the path cost begins to deviate from the optimal value in a small fraction of trials, indicating a gradual loss of the admissibility guarantee. The value α = 0.25 achieves the best balance between search efficiency and path optimality.

The local obstacle density ρ(n) is computed within a square window centered at node n. Three window sizes, 3 × 3, 5 × 5, and 7 × 7, are compared. The results are summarized in Table 5.

The 3 × 3 window, with its limited perception range, is overly sensitive to individual obstacle cells, leading to larger fluctuations in density estimation and a slight risk of path deviation from the optimum. The 7 × 7 window incurs the highest computational cost, which accumulates into a heavy burden on large-scale maps; moreover, its overly smoothed density estimation weakens the ability to discriminate local scene variations. The 5 × 5 window achieves the optimal trade-off between perception accuracy and computational efficiency.

The base look-ahead distance Db is the core reference for dynamic waypoint selection. It is tested in the range from 0.5 m to 2.0 m, and the waypoint switching frequency and total navigation time are recorded. The results are shown in Table 6.

As Db increases, the number of waypoint switches decreases monotonically. The total navigation time exhibits a U-shaped trend: when Db is too small, frequent waypoint switching forces the robot to decelerate and steer repeatedly, prolonging the navigation time; when Db is too large, the robot skips too many intermediate waypoints, causing tracking error accumulation in narrow corridors and an increase in navigation time. Db = 1.2 m yields the shortest total navigation time and a moderate waypoint switching frequency, while maintaining high trajectory smoothness, and is therefore selected as the optimal value.

To verify the robustness of the fuzzy controller to parameter perturbations, the vertex coordinates of the membership functions are randomly perturbed by ±10%, generating 10 sets of perturbed parameters. The complete navigation task is executed under each parameter set in the standard test scenario, and the total navigation time and obstacle avoidance success rate are recorded. The results are presented in Table 7.

Under all perturbed conditions, the total navigation time deviates from the nominal value by no more than 3%, and the avoidance success rate deviates by no more than three percentage points. The control surfaces exhibit negligible qualitative changes, confirming that the fuzzy controller possesses good robustness to small variations in membership function parameters.

Based on the above analysis, the optimal parameter values summarized in Table 8 are adopted in all subsequent experiments of this paper.

### 3.3. Design of Benchmark Comparison Algorithms

To fully decouple and quantify the incremental improvement effect and synergistic advantages of each innovative module in the proposed algorithm, all comparison algorithms are selected as classic baseline algorithms or mainstream improved schemes in the field of indoor service robot path planning, to ensure the fairness of experimental comparison and the scientific validity of the conclusions.

For the standalone validation of the global planning module, four groups of comparison algorithms are set up to decouple and quantify the search efficiency improvement of the proposed adaptive Octile heuristic JPS algorithm while strictly verifying the preservation of global path optimality:

- Dijkstra algorithm: the fundamental baseline for global path optimality, with no heuristic guidance, used to verify the core value of heuristic search for efficiency improvement;
- A* algorithm: the classic heuristic search baseline, adopting Euclidean distance as the heuristic function, used to quantify the redundant node elimination effect of the jump point pruning rule;
- Standard JPS algorithm: the baseline for the global module improvement in this work, adopting a fixed Octile heuristic function, used to directly quantify the incremental contribution of the proposed adaptive heuristic strategy;
- Proposed improved JPS algorithm: the experimental group, used to verify the optimization effect of the local obstacle density-based adaptive heuristic on global search efficiency.

To comprehensively validate the performance of the complete fused framework, a progressive ablation study with strict single-variable control is implemented, and a widely adopted state-of-the-art optimized DWA algorithm is simultaneously incorporated for comparative analysis, thereby systematically verifying the overall effectiveness and superiority of the algorithm proposed in this paper:

- Baseline fused algorithm: the basic verification baseline, which integrates the improved JPS and standard DWA algorithm via a traditional strategy, with no dynamic waypoint selection or fuzzy weight adjustment.
- Fused algorithm with waypoint selection strategy: this group introduces only the proposed look-ahead distance-based dynamic waypoint selection strategy on the basis of the baseline algorithm. Comparison with this group can accurately quantify the incremental contribution of the proposed global-local coordination strategy.
- Proposed complete fused algorithm: the full algorithm proposed in this work, which introduces the fuzzy control-optimized DWA algorithm on the basis of the second group. Comparison with this group can clarify the performance gain of the local planning module optimization, thus verifying the independent value and synergistic effect of each innovation point.
- PSO-DWA algorithm: a mainstream swarm intelligence-optimized DWA algorithm that uses particle swarm optimization to dynamically adjust the weights of the evaluation function.

To comprehensively and quantitatively evaluate algorithm performance, a hierarchically matched evaluation metric system is constructed in combination with the core innovation points of each module and the practical application requirements of home service robots:

- For global planning algorithm validation, the evaluation metrics include path cost, number of expanded nodes, search time, and path efficiency;
- For fused algorithm validation, the evaluation metrics include number of waypoint switches, avoidance success rate, obstacle clearance, tracking error, path length, and total navigation time.

All experiments were repeated 50 times, and the arithmetic mean of the 50 replicates was taken for each performance metric. Paired *t*-tests were used to verify the statistical significance of performance differences. A significance level of α = 0.05 was set for all statistical tests, where *p* < 0.05 indicates a statistically significant difference.

## 4. Results and Analysis

### 4.1. Feasibility Analysis of the Adaptive Octile Heuristic JPS Algorithm

To systematically verify the global planning performance of the proposed local obstacle density-based adaptive Octile heuristic JPS algorithm, mitigate stochastic interference from single experiments, and ensure the reliability and universality of the experimental results, 50 repeated global planning comparison experiments are conducted in the aforementioned typical home indoor grid map environment. The classic Dijkstra algorithm, A* algorithm, and standard JPS algorithm are selected as the control groups, and all performance evaluation metrics are taken as the arithmetic mean of the 50 repeated experiments.

**Figure 5:**
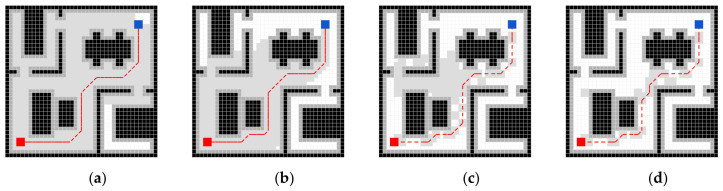
Comparison of path planning performance of different global path planning algorithms: (**a**) Dijkstra algorithm; (**b**) A* algorithm; (**c**) standard JPS algorithm; (**d**) proposed improved adaptive Octile heuristic JPS algorithm.

The visual comparison of the path search process and representative planning results of the four global path planning algorithms is presented in Figure 5. The red square denotes the navigation start point, the blue square denotes the target end point, black grids represent static obstacles in the home indoor environment, gray grids indicate the expanded nodes generated by the algorithm during the path search process, and the red line is the final planned collision-free optimal path.

It can be intuitively observed from the figure that all four algorithms can stably generate a collision-free global path from the start point to the end point, and the final output paths are experimentally observed to be optimal, which preliminarily verifies that the proposed improved JPS algorithm does not sacrifice empirical path optimality while providing a theoretical bounded-suboptimality guarantee. However, there are significant differences in the scale of expanded nodes and search efficiency among the algorithms during the search process. Specifically, the expanded nodes of the Dijkstra algorithm and A* algorithm cover most of the passable areas in the map, introducing a large number of invalid redundant searches and resulting in high overall search overhead. The standard JPS algorithm greatly compresses the scale of expanded nodes through the jump point pruning rule, effectively reduces invalid searches, and achieves a significant improvement in search efficiency compared with traditional path planning algorithms. In contrast, the proposed improved JPS algorithm has the least number of expanded nodes among the four groups, only completing the search in the key path area without redundant node expansion, which intuitively reflects the further optimization effect of the local obstacle density-based adaptive Octile heuristic strategy on path search efficiency.

The quantitative comparison results in Table 9 show that the average path cost and average path efficiency of the four algorithms are completely consistent across the 50 repeated experiments. This empirically confirms that the adaptive Octile heuristic function designed in this paper does not degrade path quality in practice, which is consistent with its theoretical bounded-suboptimality guarantee. While improving search efficiency, the algorithm maintains high stability and practical optimality under the tested conditions.

Compared with the classic baseline algorithms, the proposed improved JPS algorithm achieves a significant performance improvement. Specifically, compared with the Dijkstra algorithm, the average number of expanded nodes is reduced by 93.27%, and the average search time is shortened by 97.01%. Compared with the A* algorithm, the average number of expanded nodes is reduced by 89.24%, and the average search time is shortened by 89.70%. Compared with the standard JPS algorithm under the same framework, the average number of expanded nodes is reduced by 68.09% (*p* = 0.001), and the average search time is shortened by 52.94% (*p* = 0.001).

The global planning performance verification results for typical indoor home scenarios show that the adaptive heuristic weighting strategy based on local obstacle density can enhance the guidance to the end point in open areas of home scenarios, significantly reduce redundant node expansion, and revert to the rigorous search of the standard Octile heuristic in obstacle-dense areas. It demonstrates good adaptability to the scenario characteristics of alternating open areas and obstacle-dense areas in indoor home environments, achieves a notable reduction in global planning time compared to the standard JPS algorithm, and provides a solid experimental basis for ensuring the real-time performance of the subsequent fused navigation algorithm.

### 4.2. Feasibility Analysis of Look-Ahead Distance-Based Waypoint Strategy

To verify the optimization effect of the proposed look-ahead distance-based dynamic waypoint selection strategy on the fused algorithm, this section presents a typical indoor home navigation scenario with dynamic obstacles, based on the same grid map used in the global planning experiment. Two dynamic obstacles with fixed start and end positions and randomly fluctuating moving speeds in the range of 0.5 m/s to 1.0 m/s are added to the map for this verification.

The same global planning path, robot kinematic parameters, and navigation start and end points are adopted in the experiments, and two groups of control experiments are carried out:

- Control group: The traditional fusion strategy is adopted, which directly takes the global path jump points generated by the improved JPS algorithm as the waypoints of the local DWA algorithm;
- Experimental group: The proposed fused scheme is adopted, which takes the jump points dynamically selected based on the look-ahead distance as the waypoints of the DWA algorithm.

Both groups of experiments are repeated 50 times for verification, and all evaluation metrics take the arithmetic mean of the 50 experimental results to eliminate the random deviation of single experiments and ensure the reliability and generalization performance of the experimental results.

**Figure 6:**
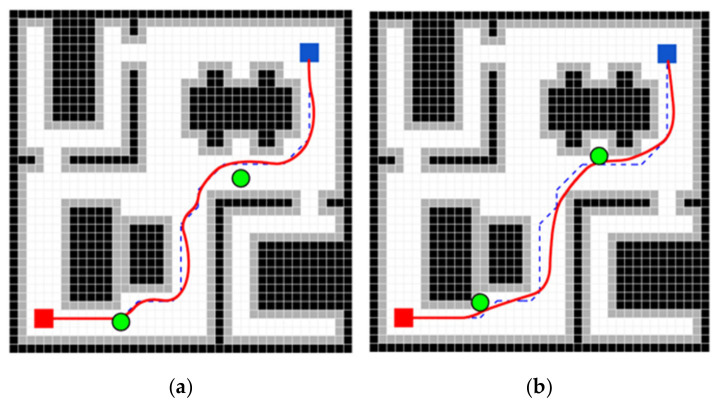
Navigation trajectory comparison before and after waypoint selection strategy optimization in dynamic scenarios. (**a**) Control group: traditional strategy with all JPS jump points as waypoints. (**b**) Experimental group: proposed look-ahead distance-based dynamic waypoint selection strategy.

Figure 6 presents the representative single planning results of the fused path planning algorithm before and after the optimization of the waypoint selection strategy, selected from 50 repeated navigation experiments. Comparing the paths planned by the two groups, it can be observed that the path of the control group has many obvious bends and jitters due to excessively frequent waypoint switching. In contrast, the path of the optimized experimental group is smooth and continuous as a whole, which can closely fit the optimal reference path generated by the global algorithm without redundant turning back and sharp turns. This verifies that the proposed dynamic selection strategy can effectively select the optimal waypoint in the trajectory planning process and realize the smooth connection between the global path and local planning.

**Figure 7:**
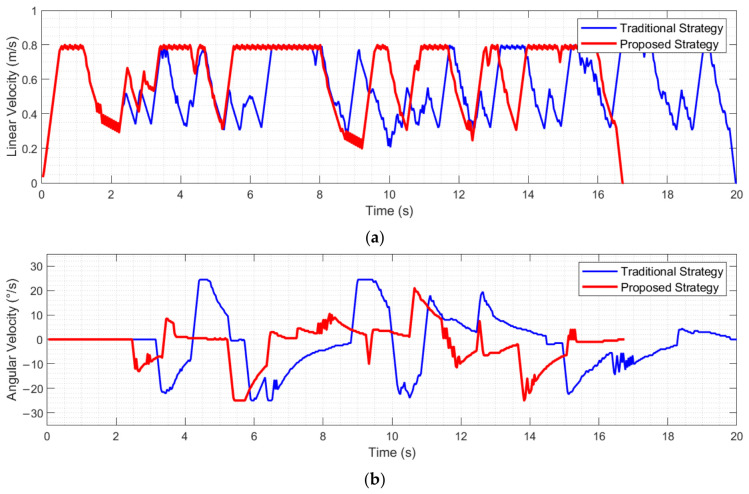
Robot motion velocity characteristics before and after waypoint selection strategy optimization: (**a**) linear velocity curves over the navigation process; (**b**) angular velocity curves over the navigation process.

It can be seen from the curve characteristics in Figure 7 that before optimization, the linear velocity curve of the robot fluctuates violently, and the angular velocity curve has many positive and negative abrupt variations. This reflects that the robot generates a large number of unnecessary acceleration, deceleration and sharp steering maneuvers due to frequent waypoint switching, resulting in poor motion smoothness and stability. After optimization, the fluctuation degree of the robot’s linear velocity curve is significantly reduced, the velocity change is more stable throughout the whole process, and the fluctuation amplitude of the angular velocity curve is greatly reduced, with a more compliant steering process. The above experimental results show that the proposed dynamic selection strategy effectively reduces redundant waypoint switching behaviors and significantly improves the velocity smoothness during the robot’s motion.

The average metrics of the 50 repeated experiments in Table 10 further quantitatively verify the comprehensive optimization effect of the look-ahead distance-based waypoint selection strategy, whose core advantages are reflected in four dimensions:

1. Significantly improved path tracking continuity: The number of waypoint switches is reduced from 18 to 7, a decrease of 61.11% (*p* = 0.001). This indicates that the dynamic waypoint selection strategy can effectively solve the inherent problems of frequent waypoint switching and redundant path correction in the traditional global-local navigation framework. By reducing unnecessary motion adjustments, the robot can follow the global path more continuously and stably.
2. Substantially enhanced navigation safety: The dynamic obstacle avoidance success rate increases from 0.70 to 0.84, corresponding to an improvement of 20.00% (*p* = 0.01) and the average obstacle clearance increases from 0.51 m to 0.57 m, an improvement of 11.76% (*p* = 0.05). These consistent improvements collectively demonstrate that the proposed look-ahead waypoint selection strategy endows the local planner with stronger forward-looking capability. Instead of reacting passively to nearby obstacles, the robot can proactively plan a safer path in advance, which effectively expands the safety margin between the robot and obstacles and reduces the risk of collision in complex dynamic environments.
3. Notably optimized navigation efficiency: The total path length is shortened from 10.94 m to 10.42 m, a reduction of 4.75% (*p* = 0.05), and the navigation time is reduced from 20.04 s to 16.74 s (*p* = 0.01), a decrease of 16.47%. This shows that the proposed strategy can avoid the detours caused by frequent waypoint switching, thus improving the overall navigation efficiency while ensuring safety.
4. Reasonable trade-off in tracking accuracy: The average tracking error increases slightly by 17.39% from 0.23 m to 0.27 m (*p* = 0.12). This is an acceptable and intentional trade-off. Unlike the traditional strategy that strictly tracks every jump point, the proposed strategy selects farther and more reasonable sub-goals, allowing the robot to move more smoothly along the global path. The tracking error of 0.27 m is still well within the acceptable range for indoor service robots.

Aiming at the core limitations of the traditional scheme that directly takes JPS jump points as DWA waypoints, including frequent waypoint switching, insufficient dynamic obstacle avoidance robustness, and low navigation efficiency, the proposed look-ahead waypoint selection strategy has been demonstrated in our simulation experiments to achieve a comprehensive and balanced optimization of path tracking continuity, navigation safety, and navigation efficiency.

### 4.3. Feasibility Analysis of Fuzzy Control Optimized DWA Algorithm

To comprehensively verify the performance of the proposed fuzzy adaptive weight DWA algorithm, three groups of controlled experiments are conducted under identical conditions. All groups share the same upper-layer improved JPS global path planning and look-ahead waypoint selection strategy, with the only variable being the local DWA variant:

- Control group: the conventional fixed-weight DWA algorithm is adopted;
- Experimental group: the proposed fuzzy adaptive weight DWA algorithm is adopted;
- Comparison group: the classic PSO-DWA contrastive algorithm is adopted.

Each group of experiments is repeated 50 times for verification to eliminate the random interference of single experiments and ensure the reliability and universality of the results.

**Figure 8:**
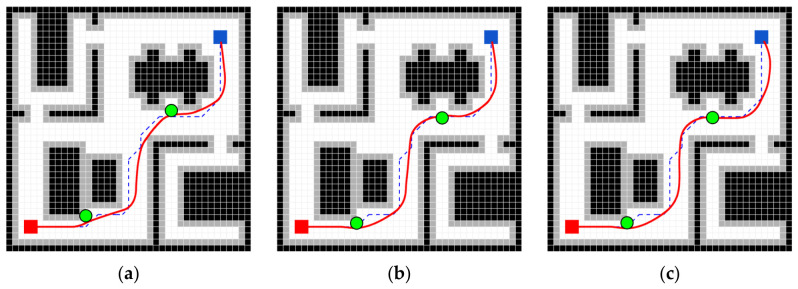
Navigation trajectory comparison of three DWA algorithms: (**a**) control group: conventional fixed-weight DWA algorithm; (**b**) experimental group: proposed fuzzy adaptive weight DWA algorithm; (**c**) comparison group: classic PSO-DWA contrastive algorithm.

Figure 8 shows representative navigation trajectories of the three DWA variants. The baseline fixed-weight DWA exhibits obvious drawbacks: frequent sharp turns, redundant offsets, and deviations from the global path, especially in corners and obstacle-dense areas. The fixed weights fail to balance obstacle avoidance safety and trajectory smoothness, often leading to abrupt steering and even local optimum traps.

In contrast, the proposed Fuzzy-DWA generates the smoothest trajectory, which is highly consistent with the global JPS path, without redundant turns or abrupt bends. The trajectory strictly conforms to the robot’s kinematic constraints, demonstrating that the fuzzy adaptive weight adjustment effectively balances obstacle avoidance safety and path tracking accuracy.

The PSO-DWA achieves improved trajectory smoothness compared to the baseline, reducing unnecessary turns and deviations, but still shows slight local offsets from the global path, and its trajectory consistency is not as good as the proposed Fuzzy-DWA.

**Figure 9:**
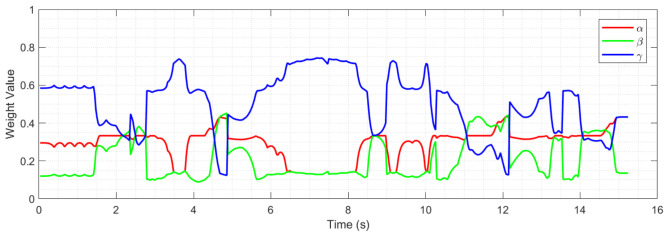
Dynamic weight variation of the fuzzy-optimized DWA evaluation function during navigation.

Figure 9 shows the dynamic variation curve of the weights of the Fuzzy-DWA evaluation function, which further reveals the adaptive optimization logic of the Fuzzy-DWA. Specifically, the algorithm collects the robot’s current motion state, local obstacle distance and density information in real time through the Fuzzy Inference System module and dynamically adjusts the weight distribution of the trajectory evaluation function. When the robot is in an obstacle-free straight section, the velocity weight γ is significantly increased, and the heading weight α is adapted synchronously, to ensure that the robot stably tracks the global path at a high speed. When the robot approaches a dynamic obstacle, the obstacle avoidance weight β rises rapidly and the velocity weight γ decreases synchronously, to give priority to ensuring obstacle avoidance safety in dynamic scenarios. After obstacle avoidance is completed, the weights quickly switch back to the high-efficiency cruise mode. This realizes the adaptive collaborative optimization of safety, tracking accuracy and navigation efficiency in dynamic scenarios and fundamentally solves the core defects of the traditional DWA, such as the inability of fixed weights to adapt to complex dynamic scenarios and the great difficulty in weight parameter tuning.

**Figure 10:**
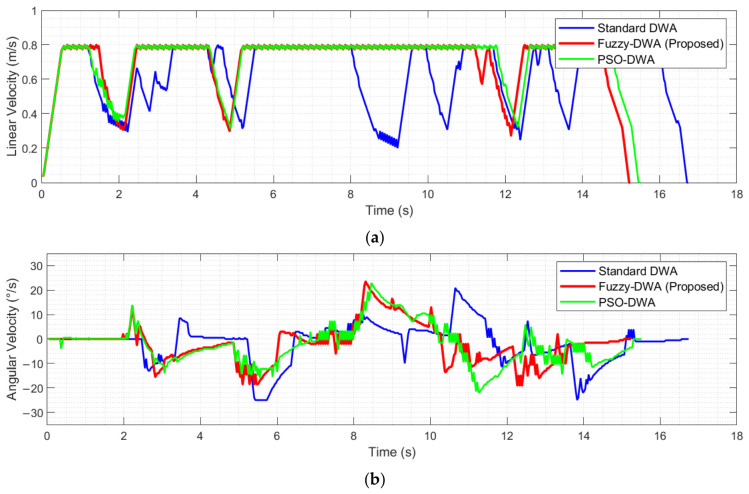
Motion characteristic comparison of three DWA algorithms: (**a**) linear velocity time-series curves over the full navigation process; (**b**) angular velocity time-series curves over the full navigation process.

It can be seen from the motion time-series curves in Figure 10 that the three algorithms show distinct differences in motion characteristics under identical conditions.

For linear velocity, the baseline fixed-weight DWA exhibits frequent sharp acceleration and deceleration, with obvious velocity oscillations and low cruising speed, reflecting its poor adaptability to alternating open and obstacle-dense areas. The proposed Fuzzy-DWA achieves the smoothest velocity profile: it maintains stable high-speed cruising in open areas, only decelerates smoothly when necessary for obstacle avoidance or path inflection, and eliminates invalid velocity oscillations. The proportion of high-speed cruising is significantly higher than the other two variants. The PSO-DWA shows improved velocity stability compared to the baseline, with fewer oscillations, but still has unnecessary decelerations and cannot maintain continuous high-speed cruising, which is inferior to the proposed Fuzzy-DWA.

For angular velocity, the baseline DWA shows violent fluctuations and frequent reverse corrections, resulting in strong steering impact. The proposed Fuzzy-DWA, however, achieves the most stable angular velocity changes, with no large reverse oscillations, and the steering process is highly matched with path curvature and obstacle avoidance requirements, effectively reducing steering impact and improving motion stability. The PSO-DWA reduces large-scale oscillations but still has occasional sharp steering corrections, and its steering stability is not as good as the proposed Fuzzy-DWA.

The average metrics of 50 repeated experiments in Table 11 further quantitatively verify the comprehensive performance advantages of the proposed fuzzy adaptive weight DWA over the standard fixed-weight DWA and the PSO-based adaptive weight DWA. All three variants are evaluated under the same look-ahead waypoint selection framework, with consistent global path planning and environmental conditions. The core improvements of the proposed Fuzzy-DWA are reflected in three dimensions:

1. Enhanced dynamic obstacle avoidance robustness: The obstacle avoidance success rate of the proposed Fuzzy-DWA reaches 0.92, which is 9.52% higher than the 0.84 of the Standard DWA (*p* = 0.01) and outperforms the 0.90 of the PSO-DWA. Meanwhile, the average obstacle clearance of the Fuzzy-DWA reaches 0.60 m, which is 5.26% higher than the Standard DWA (*p* = 0.05) and also higher than the PSO-DWA. These results collectively demonstrate that the Fuzzy-DWA achieves stronger adaptability to dynamic environments and higher navigation safety margin.
2. Optimized navigation efficiency under safety constraints: The total navigation time of the proposed Fuzzy-DWA is shortened to 15.24 s, an 8.96% reduction compared with the 16.74 s of the Standard DWA (*p* = 0.01) and also shorter than the 15.51 s of the PSO-DWA. Although the path length of the Fuzzy-DWA is slightly longer than that of the Standard DWA, this increase is not statistically significant (*p* = 0.11), and it is still shorter than the 10.95 m of the PSO-DWA. This slight increase in path length is an acceptable trade-off for higher obstacle avoidance safety, while the overall navigation efficiency is significantly improved.
3. Maintained stable path tracking accuracy: The average tracking error of the Fuzzy-DWA remains at 0.27 m, which is consistent with the Standard DWA (*p* = 1.000) and slightly better than the 0.28 m of the PSO-DWA. This indicates that the proposed fuzzy adaptive weight adjustment does not sacrifice path tracking accuracy while improving obstacle avoidance safety and navigation efficiency.

Under the unified global path planning and waypoint selection framework, the proposed Fuzzy-DWA effectively addresses the limitations of the Standard DWA, such as insufficient dynamic obstacle avoidance robustness and low navigation efficiency. Compared with the PSO-DWA, it achieves better comprehensive performance in terms of safety, efficiency and tracking accuracy, showing promising application potential for indoor service robots in dynamic complex scenarios.

## 5. Conclusions

Aiming at the core pain points of traditional fused path planning algorithms in indoor navigation scenarios of home service robots, including redundant global search, insufficient coordination between global and local planning, and poor scene adaptability, this paper proposes a path planning algorithm fusing adaptive JPS and fuzzy logic optimized DWA. The performance of the whole path planning process is improved through a hierarchical and progressive optimization design.

The core innovations and quantitative optimization effects of this paper are summarized as follows:

1. In the global planning stage, this paper designs an adaptive Octile heuristic JPS algorithm based on local obstacle density. While providing a bounded-suboptimality guarantee, the algorithm dynamically adjusts the heuristic weight to enhance guidance toward the destination and eliminate redundant node expansion in open areas while switching back to rigorous search mode in obstacle-dense regions. Experimental results show that compared with the standard JPS algorithm, the proposed algorithm reduces the average number of expanded nodes by 68.09% and the average search time by 52.94%, providing a solid foundation for the real-time performance of the hybrid navigation system.
2. In the global-local fusion stage, this paper proposes a dynamic waypoint selection strategy based on look-ahead distance. This strategy adaptively adjusts the look-ahead distance and selects the optimal waypoint according to the robot’s motion state and scene complexity, which fundamentally solves the problems of frequent waypoint switching and poor global-local coordination in traditional fusion schemes. Experimental results show that this strategy reduces the number of waypoint switches by 61.11%, improves the dynamic obstacle avoidance success rate by 20.00%, and shortens the total navigation time by 16.47%. It achieves seamless coordination between global path guidance and local planning and significantly improves path tracking consistency and navigation efficiency.
3. In the local planning stage, this paper introduces a fuzzy control module to optimize the DWA algorithm. It dynamically adjusts the weight distribution of the trajectory evaluation function based on real-time environmental information, which effectively addresses the poor scene adaptability caused by fixed weights in traditional DWA. Experimental results show that the optimized Fuzzy-DWA increases the dynamic obstacle avoidance success rate by 9.52% and shortens the total navigation time by 8.96%, while maintaining stable path tracking accuracy. Notably, it outperforms the widely used PSO-DWA in all key performance metrics, further improving the robot’s motion smoothness, dynamic obstacle avoidance robustness and navigation efficiency.

Multiple groups of comparative simulation experiments demonstrate that the proposed complete fusion algorithm achieves comprehensive optimization in four core dimensions—global planning efficiency, motion smoothness, dynamic obstacle avoidance capability, and navigation efficiency—compared with traditional fusion schemes. It is suitable for the complex indoor navigation scenarios of home environments, meets the comprehensive requirements of home service robots for navigation safety, smoothness and efficiency, and has strong engineering application value and academic reference significance. This work can also provide a new perspective for the optimization of indoor path planning technologies for home service robots.

## 6. Research Limitations and Future Work

This study has only validated the performance of the proposed algorithm through simulation experiments, and it has the following limitations:

1. All experiments were conducted in a single representative home environment scenario. The reported performance results may vary in environments with significantly different layouts or obstacle distributions.
2. The simulation environment cannot fully replicate all complex factors in real home environments, such as sensor noise, ground friction, and robot dynamic characteristics.
3. The motion patterns of dynamic obstacles adopted in the experiments are relatively simplified and differ significantly from the complex motion behaviors of real humans.

To address the above limitations, future research work will focus on the following aspects:

1. A thorough empirical analysis of the algorithm’s computational complexity will be conducted to assess its practical deploy ability on resource-constrained home service robots.
2. Realistic human motion models and sensor noise models will be introduced to enhance the realism of simulation experiments.

## Figures and Tables

**Table 1:** Core comparison between the proposed method and representative related works.

Method	Global Planning	Global-Local Coordination	Local Planning	Key Limitations
Sun et al. [8]	Density-aware heuristic JPS	Static key point selection	Standard DWA	Global density lacks local perception granularity; no dynamic waypoint selection
Guo et al. [11]	Adaptive heuristic weight	Fixed look-ahead distance	Standard DWA	Heuristic admissibility not guaranteed; waypoint selection not scene-adaptive
Wang et al. [21]	Standard JPS	Fixed waypoint	Fuzzy-DWA	Waypoint guidance not scene-adaptive; fuzzy controller inputs not coupled with global waypoint characteristics
Gong et al. [22]	Standard A*	Fixed waypoint	Fuzzy-DWA	Global planning efficiency limited by A*; no dynamic global-local coordination
Proposed Method	Adaptive heuristic based on local obstacle density	Dynamic look-ahead distance	Dual-input three-output Fuzzy-DWA	These three innovations collectively address the limitations identified in prior works

**Table 2:** Fuzzy inference rule table for weight coefficient tuning in mobile robot path planning.

Heading Deviation	Obstacle Distance	Heading Weight α	Avoidance Weight β	Velocity Weight γ
S	N	M	L	S
S	M	M	M	M
S	F	S	S	L
M	N	M	L	S
M	M	M	M	M
M	F	M	S	L
L	N	L	L	S
L	M	L	M	M
L	F	L	S	L

**Table 3:** Main motion and simulation parameters of robot.

Parameter	Symbol	Value	Unit
Maximum Linear Velocity	v_max_	0.8	m/s
Maximum Angular Velocity	ω_max_	30	°/s
Maximum Linear Acceleration	a_max_	0.8	m/s^2^
Maximum Angular Acceleration	α_max_	30	°/s^2^
Simulation Sampling Period	Δt	0.1	s

**Table 4:** Sensitivity analysis of the heuristic weighting coefficient α.

Heuristic Weight α	Global Planning Time	Expanded Nodes	Path Length (m)
0.10	31.8	42	11.12
0.20	27.5	35	11.12
**0.25**	**24**	**30**	**11.12**
0.30	22.6	27	11.57
0.40	21.5	25	11.73

**Table 5:** Sensitivity analysis of the local obstacle density window size.

Window Size	Global Planning Time (ms)	Expanded Nodes	Path Length (m)
3 × 3	31.8	35	11.34
**5 × 5**	**24**	**30**	**11.12**
7 × 7	28.5	53	11.12

**Table 6:** Sensitivity analysis of the base look-ahead distance Db.

Base Look-Ahead Distance Db	Waypoint Switches	Total Navigation Time
0.5	16	19.97
0.8	13	18.64
1.0	11	18.83
**1.2**	**7**	**16.74**
1.5	7	17.84
2.0	6	18.59

**Table 7:** Robustness analysis of the fuzzy membership function parameters.

Parameter Setting	Total Navigation Time (s)	Avoidance Success Rate
**Nominal parameters**	**15.24**	**0.92**
10 perturbed sets	15.58	0.91

**Table 8:** Summary of optimal parameter values selected based on the sensitivity analysis.

Parameter	Optimal Value	Selection Rationale
Heuristic weight coefficient α	0.25	Best balance between search efficiency and path optimality
Obstacle density window size	5 × 5	Optimal trade-off between perception accuracy and computational efficiency
Base look-ahead distance Db	1.2 m	Shortest total navigation time with moderate waypoint switching frequency
Fuzzy membership functions	Default	Performance variation <3% under ±10% perturbation, showing good robustness

**Table 9:** Performance comparison of different global path planning algorithms.

Algorithm	Path Cost	Path Length	Expanded Nodes	Search Time	Path Efficiency
Dijkstra	55.60	11.12	446	804	0.79
A*	55.60	11.12	279	233	0.79
JPS	55.60	11.12	94	51	0.79
**Improved JPS**	55.60	11.12	**30**	**24**	0.79
*p*-value	1.000	1.000	0.001	0.001	1.000

**Table 10:** Performance comparison before and after waypoint selection strategy optimization.

Item	Waypoint Switches	Avoidance Success Rate	Obstacle Clearance	Tracking Error	Path Length	Navigation Time
Traditional Strategy	18	0.70	0.51	0.23	10.94	20.04
**Proposed Strategy**	**7**	**0.84**	**0.57**	0.27	**10.42**	**16.74**
*p*-value	0.001	0.01	0.05	0.12	0.05	0.01

**Table 11:** Performance comparison of three DWA variants.

Item	Avoidance Success Rate	Obstacle Clearance	Tracking Error	Path Length	Navigation Time
Standard DWA	0.84	0.57	0.27	10.42	16.74
**Fuzzy-DWA**	**0.92**	**0.60**	0.27	10.70	**15.24**
PSO-DWA	0.90	0.59	0.28	10.95	15.51
*p*-value	0.01	0.05	1	0.11	0.01

## Data Availability

The original contributions presented in the study are included in the article; further inquiries can be directed to the corresponding author.
